# Scalp Reconstruction Using the Latissimus Dorsi Free Flap: A 12-Year Experience

**DOI:** 10.3390/jcm12082953

**Published:** 2023-04-19

**Authors:** Felix Strübing, Fabian Wenz, Nima Etminan, Amir K. Bigdeli, Laura C. Siegwart, Benjamin Thomas, Felix Vollbach, Julian Vogelpohl, Ulrich Kneser, Emre Gazyakan

**Affiliations:** 1 Department of Hand, Plastic and Reconstructive Surgery, Burn Center, BG Trauma Center Ludwigshafen, 67071 Ludwigshafen am Rhein, Germany; 2 Department of Hand and Plastic Surgery, University of Heidelberg, 69117 Heidelberg, Germany; 3 Department of Neurosurgery, University Hospital Mannheim, University of Heidelberg, 68167 Mannheim, Germany

**Keywords:** scalp reconstruction, free flap, microvascular tissue transfer, microsurgery, interdisciplinary treatment

## Abstract

Background: Complex scalp defects are regularly reconstructed using microvascular tissue transfer. The latissimus dorsi free flap is one of the workhorse flaps used in scalp reconstruction. These cases necessitate, particularly in the elderly, a close cooperation between plastic surgeons and neurosurgeons. The purpose of this study was to evaluate the suitability of the latissimus dorsi free flap for complex scalp reconstructions and to analyze potential risk factors. Methods: A retrospective study identified 43 patients undergoing complex scalp reconstruction using a latissimus dorsi free flap at our department between 2010 and 2022. Results: The mean patient age was 61 ± 18 years. Defects were mostly caused by oncologic tumor resections (*n* = 23; 55%), exposure to a cranioplasty (*n* = 10; 23%) or infection (*n* = 4; 9%). The most frequent recipient vessels were the superficial temporal artery (*n* = 28; 65%), external carotid artery (*n* = 12; 28%) and the venae comitantes (*n* = 28; 65%), external jugular vein (*n* = 6; 14%). The reconstructive success rate was 97.7%. There was one total flap loss (2%). Partial flap loss occurred in five cases (12%). Follow-up was 8 ± 12 months. Major complications were seen in 13 cases, resulting in a revision rate of 26%. Multivariate logistic regression identified active tobacco use as the only risk factor for major complications (odds ratio 8.9; *p* = 0.04). Conclusion: Reconstruction of complex scalp defects using the latissimus dorsi free flap yielded high success rates. Among the potential risk factors, active tobacco use seems to affect the outcome of complex scalp reconstructions.

## 1. Introduction

Scalp defects continue to pose a challenge to the reconstructive microsurgeon. In most cases, they result from tumor resection, in which the frequently used perioperative radiotherapy further complicates the reconstruction [1,2]. Other neurosurgical complications, such as wound healing disorders after craniotomy or implantation of cranioplasties, also make up a large portion of these cases [3,4]. Cranioplasty can be indicated for cosmetic, mechanical, and therapeutic reasons. The implantation of cranioplasties also carries a high risk of infection and can result in implant exposure in up to 7% of cases, with overall revision rates of up to 27% [3,5,6,7]. In these complex cases, after failed simple reconstructive attempts, salvage operations can only be successfully implemented in an interdisciplinary setting combining neurosurgical and microsurgical expertise. While superficial wounds may be covered by split-thickness skin grafting and smaller defects are usually amenable to local flaps, more complex defects routinely require free flap reconstructions [2]. The free latissimus dorsi (LD) flap has been used extensively for defect coverage in many regions, including the scalp [8]. The muscles’ low profile, large flap size, long pedicle, and good color matching, especially in the elderly Caucasian male, make the LD our first choice for large complex scalp defects. In addition, these patients usually have more comorbidities and are of older age compared to patients with free flap reconstructions in other anatomical regions. That is why reliable free flap procedures with low operation times are obligatory to ensure safe and sustainable results. We, therefore, evaluated the outcomes following the reconstruction of complex scalp defects using the free LD flap and examined potential outcome predictors.

## 2. Materials and Methods

### 2.1. Study Design

A retrospective review was performed on all patients undergoing free LD flap scalp reconstruction at our institution from 2010 to 2022. The institutional database for microsurgical head and neck reconstructions is prospectively maintained. The study adhered to the Declaration of Helsinki, and the study protocol was approved by the local ethics committee (medical commission Rhineland-Palatinate, Mainz, Germany, vote 2022-16297). Patients who met the inclusion criteria were selected for further analysis. The inclusion criteria were as follows: (a) full-thickness soft tissue defect with or without bone defect, (b) age > 18 years, (c) latissimus dorsi free flap transfer. Medical records were reviewed for the following parameters: patient gender, age, presenting diagnosis, comorbidities, American Society of Anesthesiologist (ASA) physical status, indication for surgery, defect location, exposed critical structures, duration of operation, operative details such as defect size, recipient vessels and type of anastomosis, outcome, surgery-associated complications, and subsequent operations. Postoperative complications were defined as follows: arterial and venous thromboses, venous congestion, wound breakdown or dehiscence, hematoma, revision for delayed or recurrent infection, partial and total flap loss, and donor site complications such as hematoma, seroma, and wound breakdown. Takebacks, partial, and total flap losses were classified as major complications. Takeback was defined as emergent surgical intervention for attempted flap salvage in case of vascular compromise. Partial flap loss was defined as tissue necrosis larger than 5 percent of the flap area requiring additional surgery in the further course. Wound healing disorders requiring surgical debridement during the first three months after flap transfer were also included.

### 2.2. Surgical Technique

All soft tissue reconstructions were performed in a similar fashion. In most cases, the patient was positioned in the lateral decubitus position, and the arm was abducted at 90° in a 3D support arm enabling free movement in all directions during the operation. The position allowed simultaneous preparation of the donor and recipient site. Only in selected cases with a concomitant neurosurgical intervention, a start in the supine position with intraoperative repositioning was necessary (*n* = 3; 7%). After the neurosurgical intervention or wound debridement, a template was fabricated. Using the template, the flap dimensions were marked. Flap elevation was performed as previously described [9,10]. A perforator-based monitoring skin island was elevated for clinical observation of flap perfusion [11].

In terms of anticoagulation therapy, 1000 IU (international units) of unfractionated heparin (UFH) were applied as an intravenous bolus prior to releasing the flap anastomoses. In case of creation of an arteriovenous loop (AVL), from 2000 to 3000 IU of UFH were applied as an intravenous bolus prior to clamping the AVL.

### 2.3. Postoperative Monitoring

All free flaps were monitored hourly for the first 48 h after surgery, followed by an evaluation every two to four hours for three days by clinical and handheld Doppler, if necessary. In total, 30 mg of low-molecular-weight heparin was administered twice daily for five days; afterward, 40 mg was administered once daily. In case of microvascular compromise, an emergent surgical revision was undertaken. Patients with increased perioperative risk or prolonged operative time were admitted postoperatively for intensive care unit (ICU) monitoring.

### 2.4. Statistical Analysis

Categorical variables are presented as frequencies (percentages) and continuous variables as means with standard deviation (SD). To identify possible risk factors for major complications, a multivariable logistic regression model was utilized. To interpret the goodness of fit, the Hosmer/Lemeshow test was used. In addition, the odds ratios (OR) with their corresponding 95% confidence intervals (CIs) were calculated. Statistical significance was defined as *p* < 0.05. Statistical analysis was performed using GraphPad Prism (version 9.0.2, San Diego, CA, USA).

## 3. Results

### 3.1. Patient Demographics

During the study period, 43 scalp reconstructions using a free LD flap were performed in our institution. The mean age of the patients was 61 ± 1 years (range, 19–88 years). Eighteen patients were 70 years or older (42%). In the entire cohort, 56% were males. The median ASA classification was three, with an interquartile range of one. The most frequent risk factor was arterial hypertension in 20 cases (47%), followed by diabetes in 11 cases (26%), and active tobacco use in 7 cases (16%). Table 1 contains further information regarding patient demographics.

**Figure 1:**
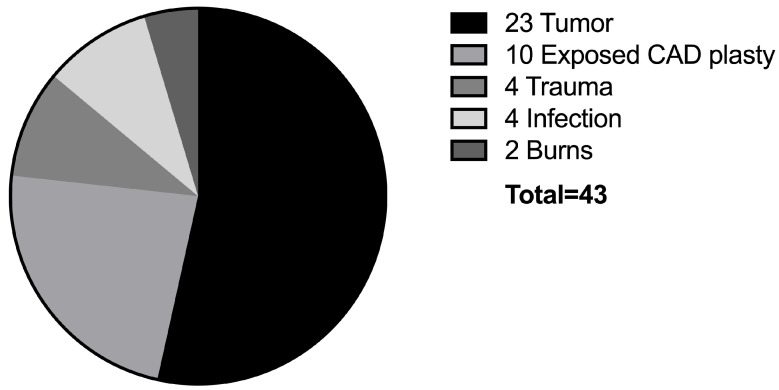
This diagram shows the defect etiology in all 43 cases of scalp reconstruction. CAD: Computer-assisted design.

**Figure 2:**
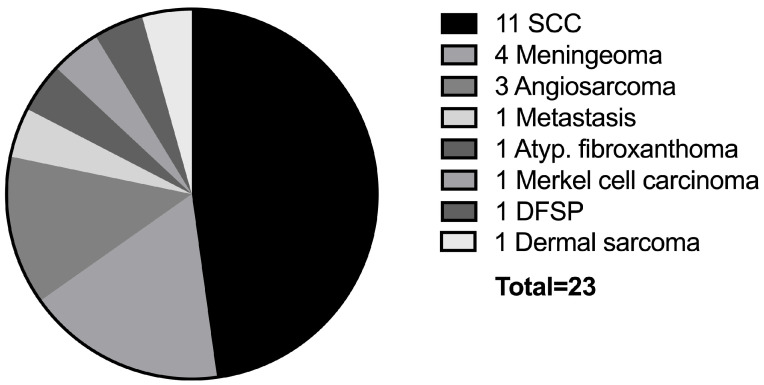
This diagram depicts the oncologic diagnosis in our tumor patients. SCC: Squamous cell carcinoma; Atyp. Fibroxanthoma: atypical fibroxanthoma DFSP: dermatofibrosarcoma protuberans.

The most common cause of scalp defects was oncologic tumor resection in 23 cases (54%). Here, the most frequent tumor was squamous cell carcinoma (*n* = 11). Exposure to a computer-aided design/computer-aided manufacturing (CAD/CAM) patient-specific skull implant was the second most common cause (*n* = 10; 22%), followed by trauma and infection (*n* = 4, respectively; 9%). Figure 1 depicts the various defect causes. The tumor entities can be found in Figure 2. The average follow-up duration was 8 ± 12 months. There was no long-term follow-up in eight cases because the patients had moved out of the catchment area if our hospital or could not be reached.

### 3.2. Operative Details

All scalp defects were reconstructed using a free LD flap. The mean operation time was 375 ± 142 min (Range: 162–693 min). In three-quarters of the cases, free flap transfer was performed in an interdisciplinary setting with neurosurgery (*n* = 32; 74%). Intraoperative repositioning of the patient, prohibiting a two-team approach, was necessary in only three cases (7%). Of the 23 oncological cases, ten patients were treated in a single-step procedure (44%), combining tumor resection and immediate microsurgical defect reconstruction. Single-stage surgery was performed if the tumor involved the inner table and dura or brain were exposed after tumor resection. Delayed reconstruction was performed in 57% of these cases (*n* = 11). There were no statistically significant differences when comparing the incidence of minor and major complications between the single-stage and delayed reconstructions (*p* = 0.58 and *p* = 0.65, respectively). The average interval between tumor resection and free flap reconstruction in these two-stage cases was 7 ± 3.7 days. In cases where preoperative imaging studies showed no infiltration of the skull and positive margins were achievable, delayed reconstructions were chosen. Single-stage reconstructions were utilized if functional structures, such as the dura mater and/or brain, were at risk of exposure or if CAD implants were used. Additionally, we employed a single-stage approach in palliative cases where complete tumor resection was not feasible and, therefore, not attempted. Complete tumor resections were achieved in thirteen cases (57%). In the eight cases with positive margins, further resection was technically not feasible or not indicated in a palliative setting. Figure 1 provides an overview over the defect etiology.

**Figure 3:**
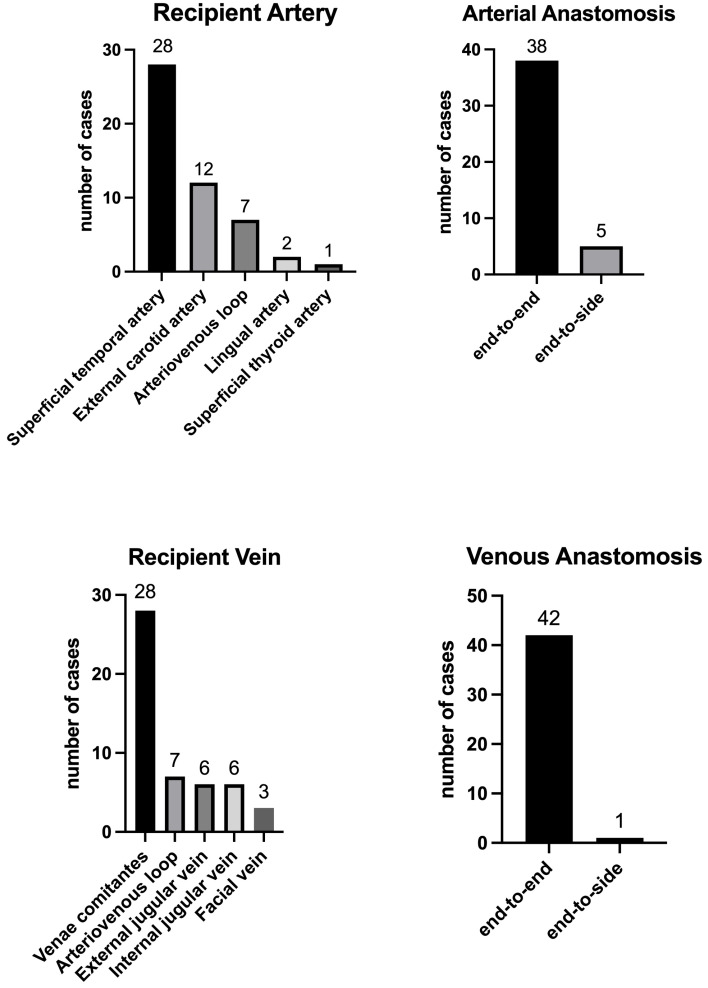
This diagram depicts the surgical details considering recipient vessels and technique of anastomosis.

For arterial anastomosis, the superficial temporal artery was chosen most frequently as the recipient vessel in 28 cases (65%), followed by the external carotid artery in seven cases (16%). An AVL was utilized in seven cases (16%), connecting the external carotid artery with the facial or jugular vein. All anastomoses to the superficial temporal, facial, and lingual arteries, as well as to the arteriovenous loops were performed in end-to-end fashion. Anastomoses to the external carotid artery were sewn in the end-to-side technique. The superficial temporal vein was most frequently used for venous drainage of the flap (*n* = 28; 65%), followed by the external jugular vein in six cases (14%) and the internal jugular vein in six cases (14%), respectively. Only one venous anastomosis was performed in an end-to-side fashion to the internal jugular vein (3%), with the rest of the cases being end-to-end anastomoses (*n* = 42; 96%). The remaining five anastomoses to the internal jugular vein were performed in an end-to-side fashion to a smaller branch dividing the main vein. Venous coupler devices were used in 21 patients (58%). Figure 3 depicts the choice of recipient vessel and anastomotic technique.

The study cohort contained 18 cases of LD flaps with perforator-based monitoring islands (42%) and four non-perforator-based monitoring islands that were debrided and skin-grafted five to seven days after the flap transfer (9%). In three cases, a pure muscle flap with any skin paddle was transferred (7%). The remaining eight cases were myocutaneous LD flaps, where the skin paddle was utilized for the definitive soft tissue reconstruction (42%). The mean defect size was 140 cm^2^ ± 86 cm^2^. The mean flap size was 309 cm^2^ ± 159 cm^2^.

Intraoperative complications were encountered in three cases (7%), and consisted of one arterial thrombosis, necessitating repeated arterial anastomosis, and one case of diffuse hemorrhage requiring prolonged hemostasis. In one case, the no-reflow phenomenon was encountered, and the flap could not be salvaged. In this case, a free vastus lateralis muscle flap was raised and successfully transferred. Twenty-one patients were admitted to the ICU postoperatively (49%) and stayed there for an average of 3.9 ± 6.2 days.

### 3.3. Flap Outcomes

There was one case of complete flap loss. Partial flap loss occurred in four cases (9%). These four cases were treated with local random pattern flaps in three patients and split-thickness skin grafting in one patient, respectively. Minor complications occurred in four cases (9%). One patient (2%) developed a seroma at the donor site that was treated with percutaneous aspiration and compression garments. Another patient developed a wound healing disorder at the recipient site, which was successfully treated by conservative wound care, and the third patient had a partial loss of the skin graft over the muscle, which was also managed conservatively.

**Figure 4:**
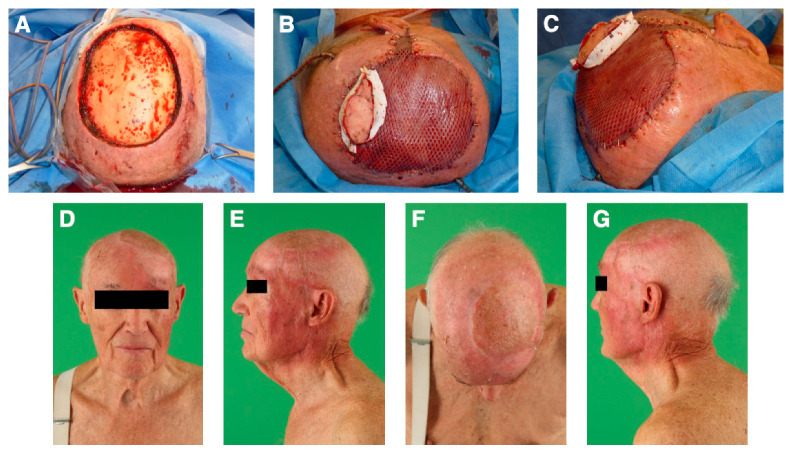
An 87-year-old male patient was diagnosed with a high-grade cutaneous angiosarcoma. (**A**): A radical tumor resection resulted in a full-thickness soft tissue defect of 11 × 11 cm. (**B**,**C**): We used a free latissimus dorsi muscle flap with a perforator-based monitoring skin paddle to reconstruct the scalp. Anastomosis was performed end-to-end to the superficial temporal artery and the superficial temporal vein. (**D**–**G**): The 18-months follow-up revealed an excellent result with a flat flap and good cosmesis. The patient had fully returned to the activities of his daily life.

There were more cases of major than minor complications (*n* = 13; 30%). In addition, to the four cases of partial flap loss, there were four cases (9%) of donor site hematoma and one case (2%) of hemorrhage on the recipient site necessitating surgical evacuations. Two patients (5%) suffered from postoperative liquor leakage, requiring surgical intervention. Infection at the recipient site occurred in two patients (5%), leading to subdural infection and osteomyelitis, respectively. One flap (2%) developed venous congestion due to kinking of the venous anastomosis and was revised successfully after emergent takeback within the first 24 h after surgery. In one case, the no-reflow phenomenon was encountered and could not be salvaged (2%). Table 2 and Table 3 depict the data on minor and major complications. Data on the number of surgical revisions can be found in Table 4. The mean interval from flap transfer to first revision was 6.7 ± 8.5 days. The second revision (*n* = 2; 5%) was performed on average 8 ± 9.6 days after the flap transfer. Figure 4 depicts an exemplary case.

Patients with incomplete tumor resections did not have significantly more major complications than those with a complete resection (*p* = 0.99). No patients deceased during their hospital stay for soft tissue reconstruction. All patients showed well-healed flaps on their latest follow-up visitations, and none of the cranioplasties had extruded.

Multivariate logistic regression revealed that the only risk factor for major complications in our cohort was active tobacco use (odds ratio 8.9, *p* = 0.04). Table 5 shows the results of the logistic regression analysis.

## 4. Discussion

In this study, we examined scalp reconstructions using the free LD flap in a single center over a period of twelve years. The presented data further solidifies the LD flap as an ideal choice for complex soft tissue defects of the scalp that require microsurgical tissue transfer. Furthermore, our results confirm the fact that extensive and complex defects are best treated by free tissue transfer.

In a retrospective analysis of a series of 892 head and neck reconstructions using microsurgical tissue transfer, Crawley and colleagues found a flap loss rate of 4.8% [12]. In the presented study cohort, we experienced similar results and encountered a partial flap necrosis rate of 9% and one complete flap loss (2%). 

Free flap reconstruction has also been shown to be safe and feasible in the elderly [13,14]. Carey and colleagues even reported the successful case of a 91-year-old patient receiving a free LD muscle flap for scalp reconstruction under local anesthesia [15]. The cohort in this study had an average age of 61 ± 18 years. Eighteen out of 43 patients (42%) were 70 years or older. Othman and colleagues reported a similar age distribution with an average age of 70.5 years in a series of 16 patients receiving free flap reconstruction of the scalp [16]. In contrast to this, the mean age of patients requiring soft tissue reconstruction of the lower extremity or in breast reconstruction is usually much lower [17,18,19,20].

Regarding microsurgical scalp reconstruction, there are many donor sites with their specific advantages and limitations [21,22]. The LD muscle flap has several significant advantages, especially for complex scalp reconstruction. It is a large flap and can be transferred as a pure muscle or myocutaneous flap. We prefer raising muscle-only flaps with subsequent skin grafting in cases without bone defects. If free flap surgery is performed after osteoclastic trepanation, we try to cover the entire bone defect with the skin paddle of the latissimus dorsi flap in order to provide some protection to the bone margins after atrophy of the muscle proportion of the flap. Because of the denervation, the muscle will atrophy over time, leading to a thinning of the flap. In this way, the flap achieves a convex contour with excellent cosmetic results in the long term. Secondary thinning procedures are, therefore, often unnecessary. A skin paddle over the muscle makes clinical monitoring easier, but it is often too bulky for definitive wound coverage. For this reason, we use perforator-based monitoring skin islands. They may be ligated and resected on the bedside after completing the monitoring phase in five to seven days [23].

The anterolateral thigh (ALT) flap is a valuable alternative to the latissimus dorsi flap. It carries similar advantages, such as a long pedicle, adequate vessel diameter, and it is possible extension to a chimeric flap with the vastus lateralis muscle or an extended fascia lata strip. However, it is considerably smaller than the latissimus dorsi flap. One significant advantage over the latissimus dorsi flap is that the ALT flap can be raised in the supine position. Possible drawbacks are its bulkiness and thus less pliability in the average Caucasian patient and its limited width. Therefore, secondary debulking procedures are regularly necessary in the follow up [21]. We use the ALT flap mainly in smaller defects or if the latissimus dorsi flap is not available.

For moderately sized to large defects, further fasciocutaneous flaps can be applied with the advantage of less donor site morbidity and lower incidence of seroma formation [24]. A more recent study evaluated a series of five thoracodorsal artery perforator (TDAP) flaps and demonstrated stable defect coverage with no postoperative complication and no flap loss [25]. Like the LD muscle flap, the TDAP flap shares the advantage of relative sparing from atherosclerosis compared to the ALT flap [26]. In contrast to other studies, we do not regularly use the radial forearm flap because of its higher donor site morbidity and the sacrifice of the radial artery [27,28].

However, the current state of knowledge continues to see the workhorses in scalp reconstructions in the LD and ALT flap [29,30]. Simunovic and colleagues evaluated both flaps in the elderly and reported no difference in the duration of hospital stay. They concluded that larger defects are best reconstructed with LD muscle flaps [13].

With regards to the recipient vessels, the superficial temporal vessels have been used in most cases due to their proximity to the defect [31]. Doscher and colleagues demonstrated in their study via radiographic analysis that the distance from the superficial temporal artery to the upper face was significantly shorter than the facial artery, enabling a more flexible flap inset [32]. In case of insufficient blood flow or small vessel diameter with a concomitant caliber mismatch, we switched to other neck vessels, such as the external carotid artery or the external jugular vein. However, this requires a long pedicle. In 16% of the cases, we successfully utilized an AVL for a better flap inset. AVLs or interposition vein grafts are still viewed critically. Maricevich and colleagues conducted a review of 241 head and neck reconstructions with interposition of vein grafts and concluded that the free flap compromise rate and free flap loss rate in vein-grafted flaps were higher compared with non–vein grafted free flaps [33]. They attributed 43.8% of the flap losses in vein-grafted flaps to surgical errors. Di Taranto and colleagues investigated 309 head and neck free flap reconstructions with an interposition vein graft and demonstrated a significant impact of graft length larger than ten centimeters on flap compromise [34]. Henn et al. analyzed a cohort of 103 AVL free flap reconstructions and found no difference between single- and double-stage reconstructions [35].

So far, there is no clear consensus on whether smoking must be considered a risk factor for microsurgical reconstruction in the head and neck region. Several authors did not find smoking to be a risk factor for flap-associated complications such as partial or complete flap loss [36,37,38]. However, a similarly large number of studies have shown that smoking might be a significant risk factor for flap loss and wound breakdown [30,39]. Considering that smoking was the only identified risk factor for major complications, we suggest additional caution in cases that involve an actively smoking patient. If elective free flap transfer is scheduled, we suggest that a strict non-smoking interval of at least one week pre- and postoperatively may be advised [40].

In our cohort, exposure to calvarial graft implants was the second most common defect cause. Here, three-quarters of the reconstructive procedures were performed in an interdisciplinary setting with neurosurgeons. In our opinion, these complex reconstructions involving soft tissue and the underlying bone make an interdisciplinary approach mandatory. These patients may also require special attention with regard to postoperative complications such as leakage of cerebrospinal fluid and neurological conspicuities. Changes in the cerebral circulation and perfusion after decompressive craniectomy due to alterations in the venous drainage and intra/extracranial pressure gradients are suspected to lead to metabolic changes and neurological impairment, also known as sinking flap syndrome or syndrome of the trephined [41]. Reviewing the literature, Annan and colleagues reported in 2015 that most patients experienced a full recovery after recallotation [41]. In our institution, cranioplasty is regularly performed.

In 2018 Gordon and colleagues published their experiences with the interdisciplinary care for patients requiring complex craniofacial reconstructions [42]. They named their approach “neuroplastic surgery”. In lower extremity reconstruction, the orthoplastic approach has been well established [43,44]. It uses periodic, interdisciplinary team meetings to facilitate a closer collaboration of orthopedic, trauma, vascular, and plastic surgeons enabling the best possible care for patients with extremity defects. We already have successfully adopted these concepts with our orthopedic colleagues [44] and plan to implement a similar strategy together with our neurosurgical service.

Although we were able to present one of the larger free-flap scalp reconstruction series, the study has some limitations. Due to the retrospective study design, we cannot rule out a selection bias or a performance bias. Because of a relatively low sample size, the study might have been underpowered to detect other risk factors than smoking. Lastly, there is some heterogeneity in the defect etiology, and we had a loss to follow up in about one-fifth of the cases. Unfortunately, we do not possess oncologic follow-up data. Nonetheless, we believe that the presented study adds relevant new insights into the application of the free LD flap for scalp reconstruction.

## 5. Conclusions

The latissimus dorsi free flap is well suited as a workhorse flap in scalp reconstruction. We found tobacco use to be a significant risk factor for major complications. Therefore, we advise at least on the week of pre- and postoperative tobacco abstinence if free flap scalp reconstruction is planned.

## Figures and Tables

**Table 1:** Patient characteristics.

Parameter	Study Population
Study cohort (*n*, %)	43 (100%)
Mean age (years, ±SD)	61.1 ± 17.6
Male gender (*n*, %)	24 (56%)
ASA classification (median, ±IQR)	3 ± 1
*Risk Factors* (*n*, %)	
Arterial hypertension	20 (47%)
Diabetes	11 (26%)
Tobbaco use	7 (16%)
Adiposity (BMI > 30 kg/m^2^)	5 (12%)
Coagulopathy	5 (12%)
PAOD	4 (9%)
History of Thrombosis/Embolism	4 (9%)

SD, standard deviation; IQR, inter quartile range; ASA, American Association of Anesthesiology; PAOD, Peripheral arterial occlusive disease.

**Table 2:** Minor complications.

Parameter	Study Population, *n* (%)
Donor site seroma	1 (2%)
Wound healing disorder	2 (5%)
Partial loss of skin graft	1 (2%)
Total number of minor complications	4 (9%)

**Table 3:** Major complications.

Parameter	Study Population, *n* (%)
Total flap loss	1 (2%)
Partial flap loss	4 (9%)
Donor site hematoma	4 (9%)
Recipient site infection	2 (5%)
Recipient site hematoma	1 (2%)
Microsurgical compromise dur to venous congestion	1 (2%)
Liquor leakage	2 (5%)
Cases with major complications	13 (30%)
Total number of major complications	16

**Table 4:** Revision surgeries.

Parameter	Study Population
Total number of revisions	16
Cases with 2 revisions	2
Cases with 3 revisions	2
Revision of donor site	7
Hematoma	6
Wound healing disorder	1
Revision of recipient site	9
Venous congestion	1
Hematoma	1
Infection	2
Wound healing disorder	3
Liquor leakage	2

**Table 5:** Prognostic risk factor for major complications.

Risk Factor	Odds Ratio	95% CI	*p* Value
Female gender	0.3	0.1–1.7	0.21
AV loop	0.5	0.1–9.8	0.67
Exposed CAD plasty	0.5	0.1–5.5	0.58
Arterial hypertension	0.5	0.1–2.4	0.32
Operating time in 75% quartile (>472 min)	0.7	0.1–6.4	0.75
Oncologic defect	0.9	0.4–6.9	0.89
Postoperative ICU admission	2.1	0.3–12.1	0.39
Diabetes	2.6	0.3–19.4	0.33
Tobacco use	8.9	1.5–93.2	0.04

## Data Availability

The data presented in this study are available on request from the corresponding author. The data are not publicly available due to the sensible nature of patient-derived information.
